# Study on dynamic characteristics of cavitation in underwater explosion with large charge

**DOI:** 10.1038/s41598-024-58622-6

**Published:** 2024-04-13

**Authors:** Jun Yu, Xian-pi Zhang, Yi Hao, Ji-Ping Chen, Yuan-Qing Xu

**Affiliations:** 1 Laboratory of Explosion Shock and Protection, China Ship Scientific Research Center, Wuxi, 214082 China; 2 Institute of Damage and Protection, Taihu Laboratory of Deep-Sea Technology Science, Wuxi, 214082 China; 3 School of Medical Technology, Beijing Institute of Technology, Beijing, 100081 China

**Keywords:** Deep-water explosion, Underwater explosion, Cavitation bubble, Phase transition, Shock wave, Cavitation collapsed, Mechanical engineering, Computational science, Fluid dynamics

## Abstract

Underwater explosions (UNDEX) generate shock waves that interact with the air–water interface and structures, leading to the occurrence of rarefaction waves and inducing cavitation phenomena. In deep-water explosions, complex coupling relationships exist between shock wave propagation, bubble motion, and cavitation evolution. The shock wave initiates the formation of cavitation, and their growth and collapse are influenced by the pressure field. The collapsing bubbles generate additional shock waves and fluid motion, affecting subsequent shock wave propagation and bubble behavior. This intricate interaction significantly impacts the hydrodynamic characteristics of deep-water explosions, including pressure distribution, density, and phase changes in the surrounding fluid. In this paper, we utilize a two-fluid phase transition model to capture the evolution of cavitation in deep-water explosions. Our numerical results demonstrate that the introduction of a two-phase vapor–liquid phase change model is necessary to accurately capture scenarios involving prominent evaporation or condensation phenomena. Furthermore, we find that the cavitation produced by the same charge under different explosion depths exhibits significant differences, as does the peak value of cavitation collapse pressure. Similarly, the cavitation produced by different charge quantities under the same explosion depth varies, and the relationship between cavitation volume and charge quantity is not a simple linear increase. The research methods and results presented in this paper provide an important reference for studying the dynamic characteristics of deep-water explosions.

## Introduction

Cavitation is a phenomenon characterized by the transformation of a liquid into vapor due to low-pressure regions in a flowing fluid^1–3^. It can have detrimental effects on the performance of lifting surfaces and the materials utilized in their construction. In the context of underwater explosions, cavitation occurs as a result of the impact of shock waves and rarefaction waves near free surfaces or solid structures^4,5^. Early simulation methods for cavitation in underwater explosions were limited by the development level of computational fluid dynamics and available computational resources. The focus was primarily on the cavitation finite element (CAFE) method^6–10^. Felippa and Deruntz employed Newton’s cavitation method based on CAFE to investigate the influence of fluid cavitation on structural response^8^. While the CAFE method provides stable predictions of cavitation in underwater explosions, its computational cost is prohibitively high for three-dimensional models. To address this issue, Sprague proposed the cavitating acoustic spectral element (CASE) method to enhance the computational efficiency of cavitation^9,10^. Subsequently, both CAFE and CASE methods have been incorporated into commercial software codes^11,12^. However, due to the limitations imposed by the theoretical assumptions of acoustic finite elements, these methods are only suitable for qualitative analysis of cavitation in the weak shock wave environment of far-field underwater explosions. Their primary advantage lies in rapidly predicting the initiation time and approximate range of cavitation, but they cannot accurately forecast the evolution process and internal characteristics of the cavitation domain^13–16^. Interface tracking methods assume the presence of a distinct interface between the liquid and vapor phases of water, employing an iterative procedure to capture this interface^17,18^. These methods provide an alternative approach to simulate cavitation in underwater explosions, allowing for a more detailed analysis of the evolution and internal characteristics of the cavitation domain.

In recent years, compressible multiphase fluid methods have gained significant popularity in the field of cavitation research^19–24^. These methods consider both the liquid and vapor phases as compressible fluids, recognizing that the phase transition between the two phases plays a crucial role in the generation and evolution of cavitation. The strong nonlinear interactions among water, explosion bubbles, cavitation, and air have a profound influence on shock wave propagation and bubble pulsation^6,19^. The cavitation domain induced by underwater explosions near a free surface occupies a large area in water and requires proper handling in simulations. Simulating the very low pressure in the cavitation zone necessitates coupling with a cavitation model. Two primary methods are adopted for simulating cavitation based on compressible fluids: one-fluid and two-fluid methods. One-fluid models have been developed to simulate cavitation, including the cut-off mode and Schmidt model^20–22^. The isentropic one-fluid model treats the fluid as a mixture comprising isentropic vapor and liquid phases^20^. The modified Schmidt model was created to improve the application of the Schmidt model in unsteady transient cavitation flow^5,23^. Another new isentropic cavitation model based on a reduced five-equation system has been proposed for researching underwater explosion cavitation^24^. However, the disadvantage of one-fluid models is their inability to capture the vapor–liquid two-phase transition during the generation of cavitation. To overcome this drawback, two-fluid models have been established in the past decade. Pelanti and Shyue developed a multiphase model based on the six-equation system, which efficiently deals with interfaces, cavitation, and evaporation waves^25,26^. A phase transition model based on the four-equation system has been used to handle the thermodynamic equilibrium between the liquid phase and its corresponding vapor phase^27–31^. This four-equation model with phase transition has been employed to investigate the evolution of cavitation in underwater explosions, enabling the capture of the physical characteristics of the flow field within the cavitation domain^13,14^. Additionally, Zhang proposed a phase transition model based on the five-equation system, incorporating temperature and chemical relaxation with the monotonic mixture speed of sound^32^. These compressible multiphase fluid methods, particularly the two-fluid models, have significantly advanced cavitation research by providing more accurate predictions of cavitation behavior and its impact on the surrounding flow field. They allow for a comprehensive analysis of phase transitions and nonlinear interactions, leading to a deeper understanding of cavitation phenomena.

In this paper, we focus on the dynamic characteristics of deep-water explosion with large charge. The objects of concern include the evolution of shock wave, explosion bubble and cavitation. The two-fluid phase transition model is used to investigate the typical dynamic characteristics of cavitation generation, development and collapse under the condition of deep-water explosion. The research results of this paper can provide an important reference for the understanding and understanding of the phenomenon of deep water explosion with large charge.

## Control equations

The 4-equation model incorporates four governing equations that capture the conservation of mass, momentum, and energy for each phase involved in the flow. These equations account for the interactions and phase transitions between the different phases, allowing for a more accurate representation of the complex dynamics in the multiphase system^28^.

1 $$\left( \begin{gathered} \rho \hfill \\ \rho u \hfill \\ \rho v \hfill \\ \rho E \hfill \\ \rho Y_{k} \hfill \\ \end{gathered} \right)_{t} + \left( \begin{gathered} \rho u \hfill \\ \rho u^{2} + p \hfill \\ \rho uv \hfill \\ \left( {\rho E + p} \right)u \hfill \\ \rho Y_{k} u \hfill \\ \end{gathered} \right)_{r} + \left( \begin{gathered} \rho v \hfill \\ \rho uv \hfill \\ \rho v^{2} + p \hfill \\ \left( {\rho E + p} \right)v \hfill \\ \rho Y_{k} v \hfill \\ \end{gathered} \right)_{z} = - \frac{n}{r}\left( \begin{gathered} \rho \hfill \\ \rho u^{2} \hfill \\ \rho uv \hfill \\ \left( {\rho E + p} \right)u \hfill \\ \rho Y_{k} u \hfill \\ \end{gathered} \right)_{r}$$

where $\rho ,u,v,p,E$ are the mixture density, the flow velocity vector in *r* and *z* directions, the mixture pressure, and the total energy per unit mass. *Y*_*k*_ denotes the mass fraction of the *k*-th phase fluid. The parameter k is assigned to each phase fluid as follows: *k* = 1 represents the liquid phase, *k* = 2 corresponds to the vapor phase associated with vapor of species 1, and *k* = 3, …, N represents other non-condensable gaseous or liquid phases. For the purposes of this paper, *Y*_3_ represents the mass fraction of air, while *Y*_4_ represents the mass fraction of explosion gas. *n* is the system parameter with values of 0, 1, or 2. In Eq. (1), if *n* = 0, it is for a 2D planar flow; if *n* = 1, it is for a 2D axis-symmetric flow with x-axis as radial direction; if *v* = 0 and *n* = 0, it is for a 1D planar flow; if *v* = 0 and *n* = 1, it is for a 1D cylindrical flow with x-axis as radial direction; if *v* = 0 and *n* = 2, it is for a 1D spherical flow with x-axis as radial direction.

To close the 4-equation model, the Noble-Abel Stiffened-Gas (NASG) equation of state (EOS) is employed. This EOS improves the accuracy of the liquid specific volume by considering repulsive molecular effects in addition to the agitation and attraction effects already accounted for in the SG EOS. This enhancement leads to better accuracy in the characterization of the liquid phase^28,33^.

2 $$\left\{ \begin{gathered} p_{k} \left( {\upsilon_{k} ,e_{k} } \right) = \frac{{\left( {\gamma_{k} - 1} \right)\left( {e_{k} - q_{k} } \right)}}{{\upsilon_{k} - b_{k} }} - \gamma_{k} p_{k}^{\infty } \hfill \\ T_{k} \left( {p_{k} ,\upsilon_{k} } \right) = \frac{{\left( {p_{k} + p_{k}^{\infty } } \right)\left( {\upsilon_{k} - b_{k} } \right)}}{{\left( {\gamma_{k} - 1} \right)C_{{{\text{v}},k}} }} \hfill \\ g_{k} \left( {p_{k} ,T_{k} } \right) = \left( {\gamma_{k} C_{{{\text{v}},k}} - q^{\prime}_{k} } \right)T_{k} - C_{{{\text{v}},k}} T_{k} \ln \frac{{T_{k}^{{\gamma_{k} }} }}{{\left( {p_{k} + p_{k}^{\infty } } \right)^{{\gamma_{k} - 1}} }} + b_{k} p_{k} + q_{k} \hfill \\ c_{k} \left( {p_{k} ,\upsilon_{k} } \right) = \sqrt {\frac{{\gamma_{k} \upsilon_{k}^{2} \left( {p_{k} + p_{k}^{\infty } } \right)}}{{\upsilon_{k} - b_{k} }}} \hfill \\ \end{gathered} \right.$$

where *e* represents the internal energy, *υ* = *1*/*ρ* represents the specific volume, *T* represents temperature, *g* represents Gibbs free energy, and *c* represents the sound speed of the phase. The Gibbs free energy, *g*, can be calculated using the formula *g* = *h*—*Ts*, where *h* denotes the enthalpy and *s* denotes the entropy. These parameters $\gamma_{k} ,p_{k}^{\infty } ,C_{{{\text{v}},k}} ,q_{k} ,q^{\prime}_{k} ,b_{k}$ are constant coefficients that reflect the distinctive thermodynamic properties of the fluid.

## Validation of one-dimensional phase transition model test

To investigate the impact of the phase transition process on cavitation and atomization formation, a one-dimensional shock tube test was conducted, as simulated by Chiapolino^28^. The test involves a mixture with initial mass fractions set to (*Y*_1_, *Y*_2_, *Y*_3_) = (0.1, 0.2, 0.7) throughout the tube. The left chamber of the shock tube is initialized with the following state variables: (*ρ*, *u*, *p*, T) = (1.941, 0, 2 × 10^5^, 354). On the other hand, the right chamber is set with (*ρ*, *u*, *p*, *T*) = (1.018, 0, 10^5^, 337.5). The fluid domain is respectively discretized into 100, 200, and 400 cells.

**Figure 1:**
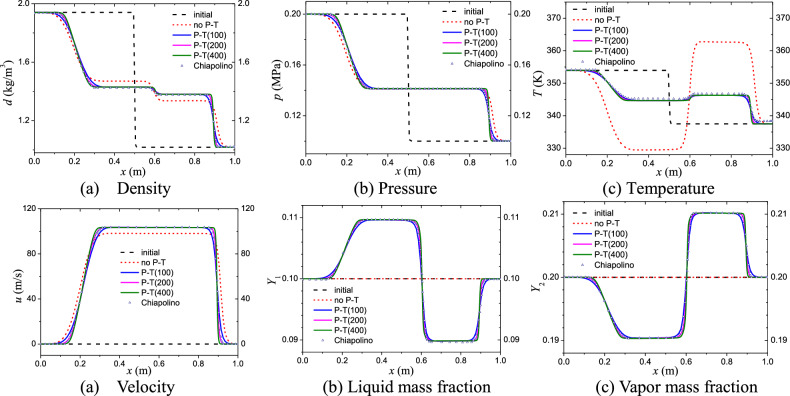
Numerical simulation results.

Figure 1 displays the density, pressure, velocity, temperature, liquid and vapor mass fraction distribution at time *t* = 1 ms. The symbols “P–T” and “no P–T" represent the results obtained with and without phase transition treatment, respectively. We can notice from the plot that there is a significant difference in the calculated results with or without phase transition. Due to the consideration of the phase transition in water, the transition from liquid phase to vapor phase occurs near the right shock wave front, resulting in an increase in the mass fraction of the gas phase and a decrease in the mass fraction of the liquid phase. As a result of the phase transition, the shock wave speed and temperature on the right side decreased. The results with 100 cells in Fig. 1 show good agreement with Chiapolino’s. We can find that the calculation accuracy increases gradually with the increase of the number of cells. In this case the calculation result using 400 cells is already very good, and the increase in calculation accuracy is not obvious if the number of cells continues to be increased.

## One thousand tons of explosives exploded at depths of 40 and 80 m underwater

**Figure 2:**
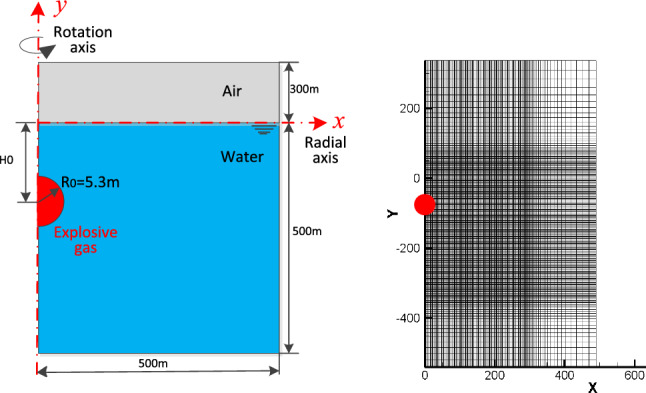
Schematic of simulation model.

To investigate the role of phase transition in the formation of cavitation and atomization in underwater explosions near the free surface, a two-dimensional axisymmetric model is utilized. The model is used to simulate the explosion generated by a 1000-ton equivalent trinitrotoluene (TNT) charge at depths of 40 m and 80 m.The 1000-ton TNT charge corresponds to an equivalent spherical radius of approximately 5.3 m (R_0_). To enhance computational efficiency, a non-uniform mesh scheme is implemented. The computational domain, as depicted in Fig. 2, extends throughout the simulation. The initial radius of the charge, denoted as R_0_, is set to 5.3 m. Within the computational domain, a uniform zone is defined with a mesh size of ∆x = ∆y = R_0_/10. This zone, measuring 40R_0_ × 80R_0_, is specifically designed to capture the motion of the explosion bubble. Impenetrable boundary conditions are enforced along the symmetric axis (y-axis), while non-reflecting boundary conditions are applied to the surrounding boundaries.

**Figure 3:**
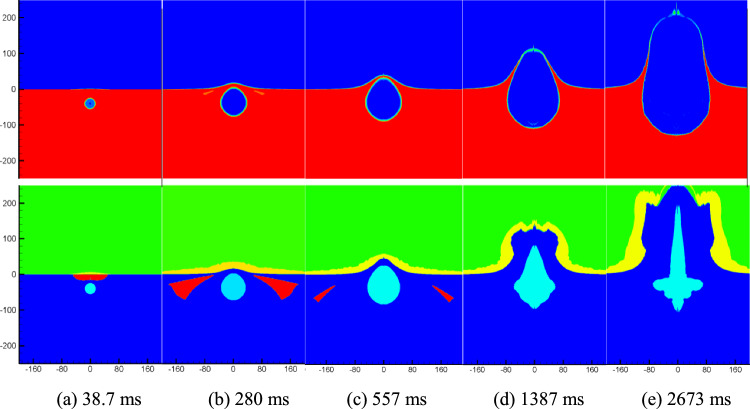
Numerical simulation results.

Figure 3 presents density and cavitation domain distribution maps in the flow field at a specific moment after the underwater explosion of a 1000 tons of equivalent TNT charge at a depth of 40 m. Upon analyzing Fig. 3, it becomes evident that at *t* = 38.7 ms, the reflected shock wave encounters the water surface, resulting in a decrease in water pressure and the initiation of cavitation. The cavitation domain primarily occupies a smaller region above the bubbles, owing to the limited area of the rarefaction wave just reflected from the water surface. Subsequently, as the shock wave continues to reflect off the water surface, the area covered by the rarefaction wave expands, causing the cavitation domain to progressively increase and spread to both sides of the explosion bubble (as observed between 120 and 478 ms in Fig. 3). By *t* = 557 ms, the cavitation domain becomes significantly thinner as the intensity of the rarefaction wave weakens and eventually disappears. At *t* = 1387 ms, the explosion bubble ruptures from the water surface, establishing a connection with the air above, forming a new gas region. This region continuously expands, giving rise to the phenomenon known as water plume. The water plume represents the upward movement of water and gas, forming a distinctive shape due to the interaction between the explosion bubble and the surrounding fluid. The analysis of the density, pressure, and cavitation distribution in Fig. 3 provides valuable insights into the complex dynamics of underwater explosions, aiding in the understanding of their effects and enabling the development of appropriate safety measures.

**Figure 4:**
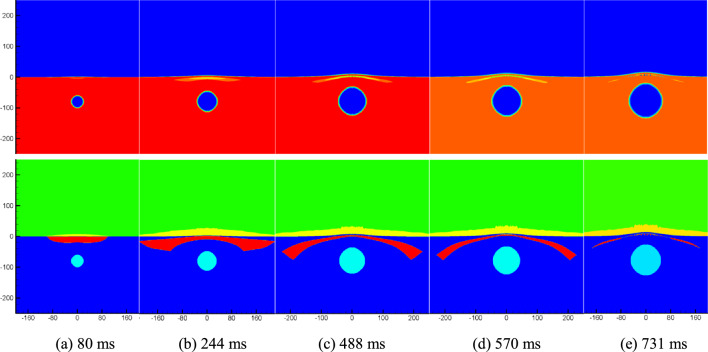
Numerical simulation results.

Figure 4 displays the density and cavitation domain evolution maps at a typical moment in the flow field following an underwater explosion of the same charge quantity at a depth of 80 m. It specifically focuses on the early-stage motion of the cavitation domain. By comparing Figs. 3 and 4, it becomes evident that as the charge depth increases, the size of the formed cavitation domain expands, but the overall thickness of the cavitation domain decreases. Additionally, it can be observed that increasing the charge depth leads to an extended duration of cavitation. The comparison between Figs. 3 and 4 provides insights into the influence of charge depth on the dynamics of cavitation domains, demonstrating the variations in their size, thickness, and duration.

**Figure 5:**
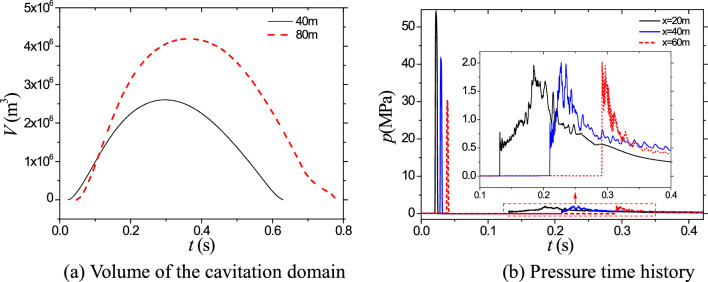
Comparison on the volume and pressure time history.

**Figure 6:**
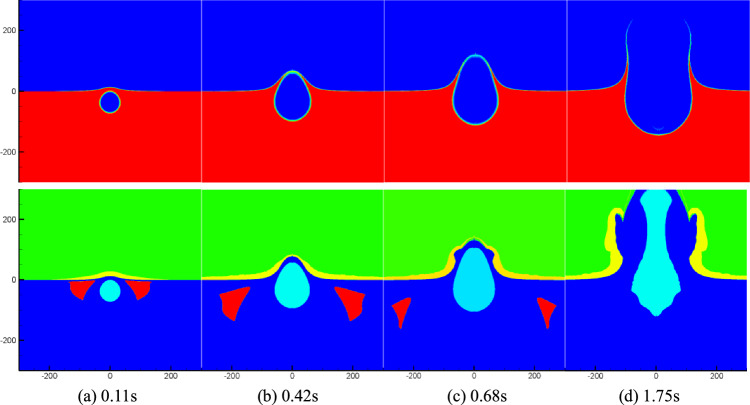
Numerical simulation results.

To provide a more intuitive comparison regarding the influence and characteristics of charge depth on cavitation evolution, Fig. 5 presents a comparative analysis of the volume variation of the cavitation domain under the two mentioned charge depth conditions. Analysis of Fig. 5a reveals that following an explosion at a depth of 40 m, the cavitation domain achieves a maximum volume of 2.6 × 10^6^ m^3^ and endures for approximately 0.63 ms. In contrast, at a depth of 80 m, these values increase to 4.2 × 10^6^ m^3^ and 0.77 ms, respectively. Consequently, doubling the water depth results in a notable 61.5% increase in the maximum volume of the cavitation domain, accompanied by a 22.2% extension in its duration. Such quantitative comparisons in Fig. 5 provide valuable insights into the impact of charge depth on the maximum volume and duration of the cavitation domain, enhancing our understanding of cavitation behavior in relation to varying water depths. Figure 6 illustrates the pressure–time curves at three measurement points located symmetrically about the axis at depths of 3 m below the water surface, specifically at distances of 20 m, 40 m, and 60 m, following an explosion at a depth of 40 m. The left side of the graph represents the shock wave pressure, while the right side corresponds to the pressure during cavitation collapse. From Fig. 5(b), it can be observed that the collapse of bubbles initiates around 0.13 ms, propagating compression waves. The peak pressure values during cavitation collapse at the two measurement points are comparable, at approximately 2.0 MPa. Although the peak pressure during bubble collapse is significantly lower than the peak pressure of the shock wave, its duration is longer and should not be overlooked due to its potential impact on structural damage in water.

## Five thousand tons of explosives exploded at depths of 40 and 80 m underwater

To further investigate the influence of charge quantity on cavitation evolution, this section simulates the explosion of a 5000 tons equivalent TNT charge at depths of 40 m and 80 m. The calculation model is similar to Fig. 2. The cylindrical explosion gas has a radius of *R*_*0*_ = 9.1 m. The rectangular computational domain for this problem is Ω = [0, 600] × [− 600, 300] m^2^, which is discretized by 1200 × 1800 mesh grids.

**Figure 7:**
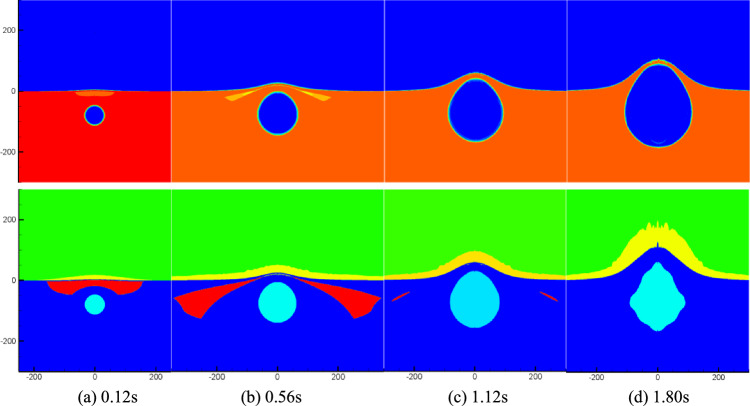
Numerical simulation results.

Figure 6 illustrates the temporal evolution of density and cavitation domain in the flow field at six representative moments following the explosion of a 5000-ton equivalent TNT charge at a depth of 40 m underwater. At time step 0.11 s, a significant number of cavitation events occur near the water surface, resulting in the formation of an annular region above the bubble (as depicted in the two-dimensional axisymmetric model). As the shock wave propagates and reflects at the free surface, the cavitation domain gradually expands, reaching its maximum volume at t = 0.42 s. Subsequently, the cavitation domain continues to move outward and expand, accompanied by the occurrence of Mizuka phenomenon from 0.42 to 0.68 s. It is important to note that due to limitations in calculation accuracy and scale, the fluid motion process could not be simulated for an extended duration. The findings from Fig. 6 provide insights into the dynamic behavior of the cavitation domain and its interaction with the surrounding flow. The temporal evolution of density, pressure, and the spatial extent of cavitation demonstrates the complex phenomena associated with underwater explosion cavitation. The observed spray phenomenon and the significant rise in water height exemplify the powerful effects of the explosion. Figure 7 presents the temporal evolution of density, pressure, and the cavitation domain in the flow field resulting from the same explosive at a representative time after the explosion, but at a greater water depth of 80 m.

**Figure 8:**
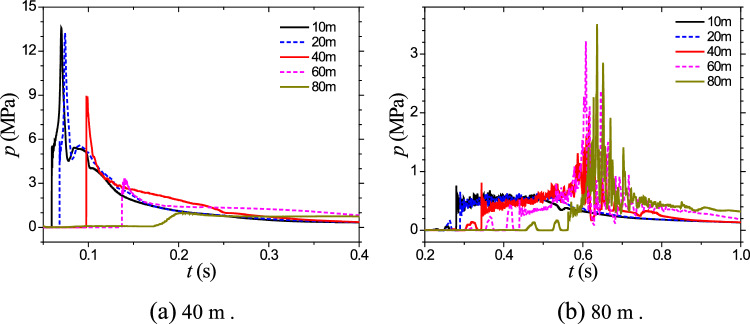
Cavitation collapse pressure induced by 5000 tons of TNT.

Figures 8 display the time history curves of pressure at a depth of 3 m from the symmetry axis for explosion depths of 40 m and 80 m, respectively. These figures provide valuable insights into the dynamic behavior of the cavitation domain, the pressure distribution, and the effects of explosion depth on cavitation evolution. The observed trends contribute to a better understanding of the complex interactions between explosive forces, water depth, and the resulting cavitation phenomena.

## Fifty thousand tons of explosives exploded at depths of 40 and 80 m underwater

**Figure 9:**
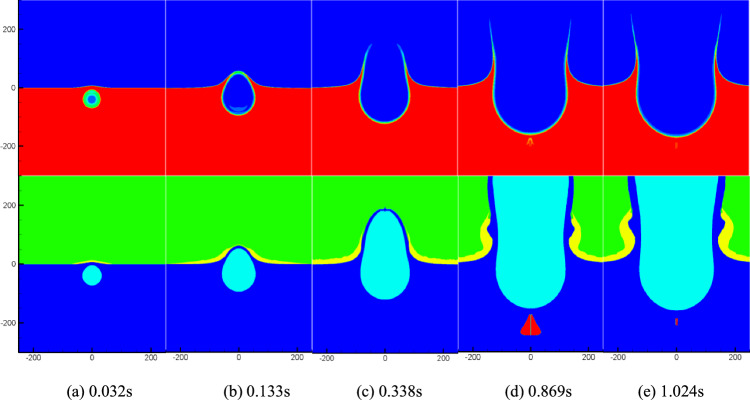
Numerical simulation results at the depth of 40 m.

**Figure 10:**
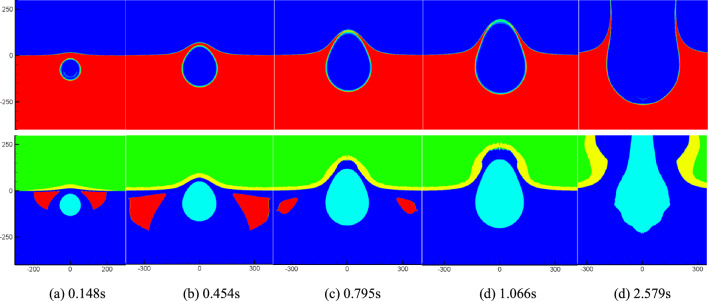
Numerical simulation results at the depth of 80 m.

In this chapter, we will further investigate the influence of charge on cavitation during the process of underwater explosion. The charge size is increased to 50 kilotons equivalent TNT, while the explosion depth remains at 40 m and 80 m underwater. The domain and boundary settings for the calculations are consistent with those in Fig. 2. Figures 9 and 10 illustrate the temporal evolution of density, pressure, and the cavitation domain in the flow field under the two detonation depth conditions, respectively. From Fig. 9, it can be observed that due to the large charge size and its proximity to the water surface, there is minimal cavitation near the water surface in the early stages. Only during the later stages of the explosion bubble movement, a very small cavitation domain appears directly below the bubble, and its duration is short. This phenomenon arises because the attenuation index of the explosion shock wave is substantial, causing the pressure to maintain higher levels after reflection on the free surface and superposition of the incident wave. As a result, it becomes challenging for cavitation to occur near the free surface. However, at tweak 0.869 s, cavitation occurs beneath the explosion bubble. This cavitation is caused by the pressure wave propagating in the flow field and being reflected on the bubble formed by the explosion. Figure 10 illustrates the temporal evolution of explosion shock waves, bubbles, and cavitation following the detonation of the same charge at a water depth of 80 m. It is evident that as the detonation depth increases, a significant amount of cavitation occurs near the water surface during the early stages of shock wave propagation. As the shock wave continues to propagate and the explosion bubble expands, the cavitation domain exhibits a vortex ring shape, gradually increasing in size while simultaneously expanding outward. The cavitation domain reaches its maximum volume around tweak 0.454 s before starting to contract. Subsequently, the explosion bubble continues to expand, leading to the formation of a water mound phenomenon on the water surface. These observations highlight the dynamic behavior of the cavitation domain, explosion shock waves, and the resulting bubble formation during underwater explosions at greater depths. The initial near-surface cavitation, followed by the expansion and contraction of the cavitation domain, contributes to a better understanding of the complex flow dynamics and the interaction between the shock wave, bubble, and cavitation phenomena. Additionally, the water mound phenomenon on the water surface demonstrates the significant impact of the explosion on the surrounding environment.

**Figure 11:**
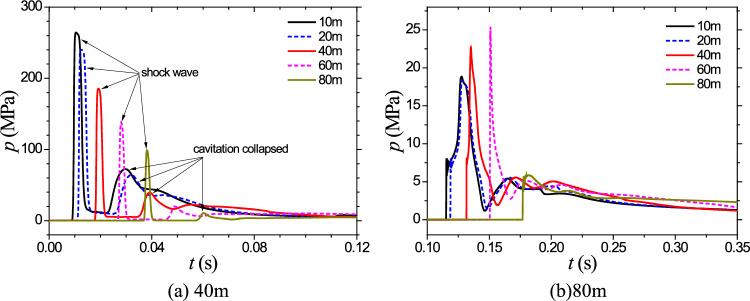
Cavitation collapse pressure induced by 50000 tons of TNT.

Figure 11a shows the temporal pressure curves at different distances from the symmetry axis at a depth of 3 m for the 40 m depth case. It can be found that due to the close proximity to the water surface and the large charge size, the pressure in the flow field after the reflection of the shock wave on the water surface is difficult to decrease below the saturation vapor pressure, making it challenging for significant cavitation to occur. Figure 11b presents the pressure curves at different measuring points for the same charge and an 80-m explosion depth. The left side represents the shock wave stage, while the right side represents the cavitation collapse stage. It can be observed that the peak pressure of cavitation exceeds 20 MPa, and its duration is also considerable, indicating that the destructive effects of cavitation should not be ignored. These findings demonstrate the variations in cavitation domain volume and the effects of explosion depth on cavitation phenomena.

**Figure 12:**
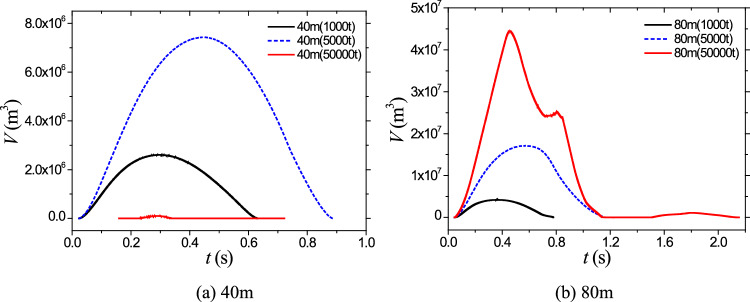
Comparison on the volume evolvement of cavitation domain.

Figure 12a provides a comparison of the volume evolution of the cavitation domain following the initiation of three different charges at a depth of 40 m. From Fig. 12a, it is evident that the cavitation volume and duration increase significantly as the charge size increases from 1000 to 5000 tons of equivalent TNT. This suggests that larger charges generate more intense cavitation phenomena with longer-lasting effects. Similarly, Fig. 12b presents a comparison of the volume evolution of the cavitation domain after the initiation of three different charges at the same 40-m depth. As shown in Fig. 12b, the volume and duration of the cavitation domain also increase noticeably with an increase in charge size. Moreover, under the condition of a 50,000-ton charge, secondary cavitation occurs, indicating a more complex and extensive cavitation phenomenon. These findings highlight the direct relationship between charge size and the volume and duration of the cavitation domain. Larger charges result in more significant cavitation effects, characterized by larger volumes and longer durations. The occurrence of secondary cavitation in the case of a 50,000-ton charge further emphasizes the complexity and potential destructive nature of cavitation phenomena in underwater explosions.

## Conclusions

The study used a two-fluid phase transition model to investigate cavitation under deep-water explosion conditions. Various hydrodynamic characteristics of cavitation were examined for different charge sizes and explosion depths. The key conclusions are as follows:

1. The phase change model significantly influenced the flow field's density, pressure, and phase distribution, requiring a two-phase vapor–liquid model for accurate representation.
2. The model effectively captured cavitation generation, development, and evolution during deep-water explosions, with cavitation domains initially exhibiting an annular shape and expanding outward before collapsing.
3. Cavitation duration and volume varied with charge size and explosion depth, with noticeable increases observed as the explosion depth increased. The peak pressures during cavitation collapse were within certain limits for different charge sizes, but the 50,000-ton charge exhibited higher pressures and secondary cavitation in some cases.

The findings of this study are primarily based on limited data from deep-water explosions involving large charge sizes. Further extensive research, including three-dimensional modeling, fluid–structure interaction analysis, adaptive mesh refinement, and consideration of detonation reactions, is warranted to apply these results to practical engineering applications.

## Data Availability

The data that support the finding of this study are available from the corresponding author upon reasonable request.
